# Do environmental degradation and geopolitical risk hinder life expectancy? The role of institutional quality and technological innovation

**DOI:** 10.3389/fpubh.2025.1650912

**Published:** 2025-12-10

**Authors:** Yunqiu Zhan, Hao Yin, Xiaolan Zhang

**Affiliations:** 1 Chengdu Technological University, Chengdu, China; 2 Xihua University, Chengdu, China; 3 The First Affiliated Hospital of Zhejiang Chinese Medical University (Zhejiang Provincial Hospital of Chinese Medicine), Hangzhou, China

**Keywords:** geopolitical risk, CO2 emissions, institutional quality, life expectancy, BRICS, CS-ARDL

## Abstract

Environmental degradation (ED) and geopolitical risk (GPR) reduce life expectancy (LE) by worsening health through pollution and ecosystem damage, and by disrupting healthcare systems and basic services via conflicts, displacement, and food insecurity. Together, they create unstable living conditions that shorten lifespans. Thus, this study aims to investigate the impact of ED, GPR, institutional quality (IQ) and technological innovation (TI) on LE in BRIC economies from 1993 to 2022. This study employed the second-generation econometrics technique. We used Cross-sectional augmented distributed lag (CS-ARDL), fully modified ordinary least squares (FMOLS), and Dynamic ordinary least squares (DOLS) methods. The result shows that ED and GPR have negative effect on LE, while IQ, TI and GDP have positive effect on LE. These findings present enormous contributions, significant research implications to BRICS nations and development literature, and policy implications for LE of BRICS economies.

## 1 Introduction

Health is a central focus of SDG 3, which seeks to ensure healthy lives and promote well-being for people of all ages. This goal emphasizes reducing maternal and child mortality, combating diseases, and enhancing healthcare access. Additionally, health plays a vital role in supporting other SDGs, such as poverty reduction and quality education, by contributing to more productive populations. Across the globe, individuals need improved healthcare facilities, and nations work toward providing better health services and related amenities for their citizens (1). Life expectancy (LE) is a key indicator of a country’s overall health. “*LE at birth estimates the number of years a newborn is expected to live, assuming that the mortality rates at the time of birth remain unchanged throughout their lifetime*” (2). Enhancing LE is a key goal for countries worldwide as they strive to improve the health of their citizens. To achieve this, they focus on reducing mortality rates and enhancing overall health conditions.

Climate change (CC) is widely regarded as one of the biggest threats to future population health and global development (3). Its effects on population health are diverse and substantial (4). The World Health Organization (5) estimates that 4.2 million premature deaths worldwide were due to air pollution in 2016. As nine out of 10 people live in places with poor air quality, is anticipated to climb. People’s health may be harmed by environmental degradation in several ways, and it has been related to severe air pollution. According to Majeed and Ozturk (6) environmental deterioration affects population health in multiple ways, including thermal stress due to CC, the spread of vector-borne diseases such as malaria and dengue fever, and unfavorable changes in food production. It also leads to higher concentrations of outdoor air pollutants, contributing to chronic illnesses like asthma, heart disease, and lung cancer, as well as increasing the risk of premature mortality. Additionally, environmental degradation can trigger extreme weather events, elevate exposure to aeroallergens, and promote the spread of waterborne diseases and other infectious illnesses. The nexus between environmental degradation and LE were examined by Uddin et al. (7) for SAARC, Azam et al. (8) for Pakistan, Mahalik et al. (9) for BRICS, Dam et al. (10) for BRICS- T, all of these found that environmental degradation had negative effect on LE.

Over the past few decades, geopolitical risk (GPR) has escalated in practically every region of the world. There have been conflicts between nations, terrorist attacks, and wars around the world. GPR also affects society, politics, health and the economy. Furthermore, a few geopolitical events, such as the trade war between the US and China trade war, 9/11, and the bombing of Bombay, have altered the attitudes and actions of economic actors. GPR is seen by investors, businesses, and central banks as a crucial factor in determining economic performance (11, 12). Numerous research demonstrate how GPR has an economic impact. According to Saint Akadiri et al. (13), GPR has a detrimental impact on economic growth and tourism. Similarly, GPR has an impact on tourist receipts, according to Alola et al. (14) and Athari et al. (15). While Su et al. (16) show that GPR influences oil prices and financial liquidity, Rasoulinezhad et al. (74) discover that GPR affects energy usage. Furthermore, according to Pan (17), GPR reduces R&D spending. Wang et al. (18) also come to the conclusion that investments and GPR have a bad association. Olanipekun and Alola (19) have discovered that increases in geopolitical threats have a negative impact on oil output. Furthermore, Pehlivanoğlu et al. (20) elaborate on the causal link that exists between producer, consumer, and geopolitical risk confidence. Dogan et al. (21) reported that GPR discourage the natural resources. Yu and Wang (22) found that GPR significantly reduced the FDI and trade. Jha et al. (23) found that advanced economies are better equipped to withstand GPR shocks and enjoy favorable rates of development. Conversely, developing economies assert that geopolitical risk has a negative influence on their economic growth. Some scholar reported that GPR have also effect the environment. According to Anser et al. (11) and Cui et al. (24) reported that GPR discourage the environmental quality in BRICS economies. Paramati et al. (25) reported that GPR raises carbon emissions and these two together amplify public health risks.

In the previous studies on the link between GPR and LE, several proxies of GPR (e.g., war, terrorism, political instability) have been used. Plümper and Neumayer (26) reported that on average, women are more negatively impacted by interstate and civil conflicts than males are over the course of the conflict. Women usually live longer than males do during times of calm. Therefore, the difference in LE between men and women tends to close during armed conflict. They also discover that, in comparison to previous civil wars, ethnic conflicts and battles fought in “failed” governments cause significantly greater harm to women. Mahmud (27) found that terrorism has negative effect on human development. Feyzabadi et al. (28) found that peace index had negative effect on LE. Wesemann et al. (29) found that the prevalence rates of major depression, anxiety disorders, and post-traumatic stress disorder ranged from 1.3 to 25.8%, 1.7 to 14%, and 1.3 to 16.5%, respectively. The World Trade Center attacks were associated with the greatest prevalence rates. Gender, lack of emergency services training, peritraumatic dissociation, physical closeness to the incident, and social isolation were all listed as risk factors. Tin et al. (75) found that between 1970 and 2019, South Asia was the site of 23.69% of all terrorist incidents worldwide, leading to 96,092 deaths and 141,333 non-fatal injuries. Among these incidents, explosives were the most commonly used weapon, responsible for 50.1% of attacks, followed by firearms (31.9%), unknown weapons (9.4%), incendiary devices (5.9%), melee weapons (2.3%), and chemical, biological, or other weapon types (less than 0.5%).

Numerous studies have empirically demonstrated the significant impact of institutional measures on health-related outcomes (76, 77). Rehmat et al. (76) found that lower institutional quality is associated with shorter life expectancy, whereas strong institutional quality positively influences LE (7). Additionally, anecdotal arguments suggest that differences in institutional quality help explain the life expectancy gap between developed and developing nations (30). Technological innovation (TI) enhances LE by improving healthcare access, advancing medical treatments, and reducing disease burdens. Clean technologies and sustainable energy lower environmental hazards, leading to better overall health. Also, digitalization facilitates efficient healthcare delivery, early diagnosis, and disease prevention. Jiang et al. (31) examined the impact of digitalization and clean technologies on BRICS countries, finding that green technology positively affects long-term LE in Russia and China, though its short-term influence on health outcomes is minimal. Their study revealed that a 1% increase in clean technology usage led to a 0.125% rise in life expectancy in Russia and China, while in South Africa, the increase was 0.008%. The adoption of sustainable energy sources has been shown to mitigate the adverse health effects of traditional energy production, reducing heat-related population impacts and improving overall health outcomes, including lifespan and fatality rates (32).

The reason for selecting the BRICS economies is that the BRICS countries represent nearly 40% of the world’s population. Together, they account for about 25–27% of global GDP and over 20% of global trade. China and India, with their massive populations, are the primary drivers of demographic weight. Economically, China contributes the largest share, followed by India, Russia, Brazil, and South Africa. Collectively, BRICS play a pivotal role in shaping global economic growth and development. According to IEA data, the BRICS countries collectively contribute more than 40% of global CO₂ emissions, making their mitigation pathways crucial for realizing the temperature targets of the Paris Agreement. Rapid economic growth and accelerated urbanization in these economies have driven rising energy demand and continuous industrial upgrading, which in turn fuel the upward trajectory of emissions. Nevertheless, notable differences remain across BRICS nations with respect to their levels of economic development, energy mixes, industrial structures, and the adoption of emission-reduction technologies (33).

Building on the significance of the aforementioned variables, this study explores their impact on LE. Specifically, it integrates CO2-E, GPR, IQ, and technological innovation factors that have not yet been comprehensively examined in previous empirical research on BRIC countries. By incorporating these key determinants, this study contributes to filling a critical gap in the literature and enhancing the understanding of their combined influence on public health and longevity. Accordingly, this research focuses on the following objectives:

1. To examine the effect of air pollution, particularly CO2-E on life expectancy
2. To investigate the impact of geopolitical risk on life expectancy.
3. To analyze the role of institutional quality and technological innovation in shaping life expectancy.

By addressing these objectives, this study provides new insights into the complex interactions between environmental, political, institutional, and technological factors influencing life expectancy in BRIC nations.

The primary objective of this empirical study is to assess the impact of CO2-E, GPR, IQ, and technological innovation on LE across four BRIC nations. By incorporating these critical factors, this research seeks to fill an existing gap in the literature and provide a more comprehensive understanding of the determinants of longevity. This study makes five key contributions to the existing body of research: first, unlike previous studies that primarily focused on war and terrorism as isolated variables, this research explicitly examines the effect of the GPR index on LE. The GPR index captures a broader spectrum of geopolitical uncertainties, including political conflicts, trade tensions, and global instability, which can significantly influence healthcare systems, economic growth, and overall public well-being. Second, this study concurrently investigates the effects of CO2-E, GPR, IQ, and TI on LE, a comprehensive approach that has not been previously explored in BRIC countries or even globally. Understanding the interplay of these factors is crucial for designing effective policies aimed at improving life expectancy. The inclusion of CO2-E GPR, IQ, and TI recognizes the multidimensional nature of life expectancy determinants. Developing effective strategies to enhance public health requires an in-depth analysis of these factors, yet prior studies have largely ignored their combined influence. By quantifying their individual and collective impact, this research offers valuable insights for policymakers. Third, In addition to the primary variables of interest, this study incorporates key control variables such as GDP, ensuring a more robust analysis by accounting for economic factors that influence health outcomes. The inclusion of GDP provides a clearer picture of how economic development interacts with life expectancy determinants. Fourth, unlike prior research that relied on traditional econometric approaches, this study employs second-generation econometric techniques to address issues of heterogeneity and cross-sectional dependency. These methodologies enhance the accuracy and reliability of the findings, offering a more sophisticated analysis of the relationships between the examined variables. By investigating the determinants of life expectancy within the BRIC context, this study makes a valuable contribution to the literature, providing fresh insights for scholars and policymakers. The findings will help shape effective strategies aimed at enhancing public health and longevity in BRIC nations and beyond.

The remainder of this article is structured as follows: Section 2 presents the literature review, Section 3 outlines the methodology and data, Section 4 discusses the results and findings, and Section 5 provides the conclusion along with policy recommendations.

## 2 Literature review

### 2.1 Theoretical review

One of the most important components of human capital is health, which is seen as a long-lasting characteristic that has a favorable correlation with labor productivity. Grossman (34) offered the basic theoretical framework in the literature for modeling health. The suggested health production function is regarded as one of the most important instruments for examining the health status of a nation. The classic production function, which illustrates the relationship between socioeconomic input and output, is comparable to the health production function. Reduced morbidity or LE is used to assess the dependent variables of the health production function; on the other hand, income, education, health care, the environment, health and medical expenditures, genetic characteristics, etc. are the inputs (7, 35).

The Preston (36) Curve describes the positive relationship between a country’s income level and its average LE. At lower income levels, small increases in income lead to significant gains in LE due to better nutrition, healthcare, and living conditions. However, as income rises, the curve flattens, meaning additional income brings smaller improvements in LE. This theory highlights how economic growth contributes to health outcomes, but with diminishing returns at higher income levels. This theory is also validated by Rahman et al. (37) in 31 of the world’s most polluted countries and Alhassan et al. (38) in Developing Countries.

Human health is impacted by political, social, and economic instability and upheavals (riots, civil wars, and conflicts) due to the devastation caused by various weapons, as well as the pressures and punishments imposed by the social, political, and economic structures. People discuss their health requirements throughout this era, as well as the ensuing malnutrition, drought, and diseases caused by these disturbances, as well as the misallocation of financial resources to military purposes (39, 40). Changes in death trends and an increase in suicides may result from economic stress and insecurity (41, 42). Donors’ technical and financial assistance as well as peacekeeping efforts during various unrests and instabilities can have an operational impact on the health sector (43). According to Majeed and Ozturk (6) categories into which climate change influences health into three categories such as direct, indirect, and delayed consequences. The deaths and injuries brought on by extreme weather events like floods and cyclones are among the direct or major effect. The infectious illnesses brought on by climate change are among the indirect or secondary impacts. Disruption to social and health services is one of the delayed or tertiary consequences, which develops gradually (44, 45).

### 2.2 Relationship between environmental degradation and health

Hossain et al. (46) analyzed the connection between CO2-E and LE in Bangladesh for the period from 1974 to 2024 by using the ARDL approach. They found that environmental deterioration has a major detrimental effect on life expectancy over the long term. Ali and Audi (47) analyzed CO2-E, income inequality and LE in Pakistan from 1980 to 2015 by using the ARDL approach. The study’s findings indicated that Pakistan’s life expectancy is significantly impacted negatively by both environmental deterioration and income disparity. Das and Debanth (48) analyzed Impact of CO2-E on LE in India from 1991 to 2018 by using the ARDL methods. According to the study finding CO2-E has negatively affect LE. Using the ARDL model, Osabohien et al. (49) examined the relationship between life expectancy and carbon emissions in Nigeria from 1980 to 2017. The results indicated that life expectancy is negatively impacted by carbon emissions. This result suggested that life expectancy can be lowered by 0.35% on average due to carbon emissions. Azam et al. (8) investigated the connection between CO2-E and LE in Pakistan for the period from 1975 to 2020 by utilizing the ARDL approaches. They concluded that, management authorities must control carbon emissions to increase life expectancy, which is a major factor in determining economic growth. Azam and Adeleye (50) investigated the association between CO2-E sources and LE in in Asia and the Pacific region from 2005 to 2010. They found that CO2-E has negative effect on LE. Guo et al. (51) analyzed SAARC countries from 1990 to 2022 using the panel ARDL model to capture both short- and long-run effects, complemented by the method of moments quantile regression (MMQR) to explore heterogeneity across quantiles. Their results revealed that CO₂ emissions reduce life expectancy and increase infant mortality, while renewable energy, urbanization, GDP, and industrialization improve health outcomes. To ensure robustness, they applied FMOLS and DOLS estimators, which further validated the main findings.

### 2.3 Relationship between geopolitical risk and health

Previous studies examining the relationship between geopolitical risks and LE have employed various proxies for geopolitical risks, such as war, terrorism, and political instability. Plümper and Neumayer (26) examined the Armed Conflict’s Impact on the Gender Difference in Life Expectancy. This paper offers the first thorough examination of how armed conflict affects women’s life expectancy in comparison to men’s. We find that, on average, women are more negatively impacted by interstate and civil conflicts than males are over the course of the conflict. Women usually live longer than males do during times of calm. Therefore, the difference in life expectancy between men and women tends to close during armed conflict. We also discover that, in comparison to previous civil wars, ethnic conflicts and battles fought in “failed” governments cause significantly greater harm to women. Mahmud (27) analyzed the nexus between terrorism and human development in Iraq, they found that terrorism has negative effect on human development. Feyzabadi et al. (28) examine the nexus between peace and LE among world countries from 2007 to 2012 by using the fixed effect. They found that peace index had negative effect on LE. Wesemann et al. (29) analyzed the relationship between terrorist attacks and mental health based on Quantitative Studies. They found that the prevalence rates of major depression, anxiety disorders, and post-traumatic stress disorder ranged from 1.3 to 25.8%, 1.7 to 14%, and 1.3 to 16.5%, respectively. The World Trade Center attacks were associated with the greatest prevalence rates. Gender, lack of emergency services training, peritraumatic dissociation, physical closeness to the incident, and social isolation were all listed as risk factors.

Chukwuma (52) examined the geopolitical dimensions of health and biosecurity in relation to gain-of-function research. The study highlighted widespread global concerns about how emerging and reemerging infectious diseases spread, as well as the geopolitical implications tied to the nature of gain-of-function research. These issues have become increasingly important for both national and international health governance, as infectious diseases are clearly geopolitical in nature. The research emphasized that health diplomacy now plays a critical role in addressing current and future outbreaks, pandemics, and microbiome variations linked to gain-of-function studies. Towne (53) used logistic regression to assess geopolitical disparities in US health care access from 2011 to 2015. Research, practice, and policy related to connecting people to essential medical care are informed by the conclusion that geopolitical factors state Medicaid expansion or not may influence whether or not an individual reports forgoing medical treatment. Paramati et al. (25) investigated the impact of geopolitical risk on CO₂ emissions and public health risk across 17 countries during 1990–2018. Using generalized quantile regression and panel corrected standard errors, their results confirmed that geopolitical risk amplifies carbon emissions, which subsequently escalates health risks. Their robust methodology strengthens the evidence that political instability indirectly undermines public health through environmental degradation.

### 2.4 Relationship between institutional quality and health

Badmus et al. (54) explored the role of IQ in Health Expenditure in Africa by using the GMM technique. They discovered that the crowding-out impact on government health expenditures in Africa is only reversed when an interaction term including military spending and institutional quality is included; private and out-of-pocket health expenditures remain unaffected. In a similar vein, the interaction term’s positive impact on health outcomes is limited to higher life expectancy and lower rates of maternal death. Sharma (55) analyzed IQ and health outcomes in European Union countries from 2000 to 2018 by using the GMM technique. The results suggest a positive association between the quality of economic institutions and health outcomes. Hadipour (56) analyzed the IQ and health outcomes nexus for 158 countries from 2001 to 2020 by using the GMM methods. The findings showed that while institutional quality increased life expectancy, it decreased newborn mortality rates. Similar correlations were found between newborn mortality rates and life expectancy and factors including GDP, mean years of schooling, total health spending, and urbanization rate. Nica et al. (57) analyzed IQ and LE nexus in Eastern Europe from 1990 to 2021 by using the CS- ARDL methods. They found that IQ encourage to surge the LE. Ejuvbekpokpo (35) analyzed the impact of IQ on human development in SSA countries for the period from 2005 to 2013 by using the GMM methods. The findings demonstrate a relationship between poor levels of human development and institutional quality. However, the findings indicate that, in the SSA nations, institutional quality is critical to human development. Uddin et al. (12) examined the connection between IQ and LE in SAARC economies from 2002 to 2020 by using the CS-ARDL methods. They found that IQ rise the LE. In the context of developed economies, Vatamanu et al. (58) explored the relationship between renewable energy, institutional quality, and life expectancy in 27 EU countries from 2000 to 2020. Employing cointegration analysis, factor analysis, panel FMOLS tests, they found that renewable energy consumption and institutional quality both significantly enhance life expectancy. Their findings highlight that strong governance institutions amplify the effectiveness of renewable energy adoption in sustaining longevity and improving overall well-being.

### 2.5 Relationship between technological innovation and health

Segbefia et al. (59) examined the effects of renewable energy, TI, CO2-E, and human capital on LE in NAFTA economies. Utilizing the CS-ARDL model and panel data from 1990 to 2020, the study focuses on the United States, Mexico, and Canada. Findings indicate that human capital, renewable energy, technological innovation, and economic growth positively affect LE, while carbon emissions have a negative impact. Causality tests reveal a unidirectional relationship between human capital, economic growth, technological innovation, and LE, and a bidirectional relationship between carbon emissions, renewable energy, and LE. The study recommends stringent air quality regulations and cleaner technologies to enhance LE. Shaari et al. (60) investigated the impact of green technology, CO2-E, and health expenditures on LE in ASEAN-5 countries by using the FMOLS and DOLS estimators and CS-ARDL, the study focuses on ASEAN-5 (Thailand, Philippines, Singapore, Malaysia, and Indonesia). Findings indicate that green technology significantly enhances LE, while CO2 emissions negatively affect it. FMOLS and DOLS results highlight that health expenditure improves LE in Thailand and the Philippines. Economic expansion positively influences LE in Thailand, the Philippines, and Singapore. The study recommends prioritizing healthcare investments, promoting clean energy, and implementing incentives for green technology to enhance economic resilience and public health. Muradov et al. (61) analyzed the relationship between air pollution and LE in the United States, considering medical innovation, health expenditures, economic complexity, and government effectiveness. Using cointegration analysis and data from 1995 to 2019, the study finds that medical innovation positively affects LE, while CO2 emissions, economic complexity, and government effectiveness negatively impact it. Health expenditures are found to be ineffective in improving LE. The study suggests that policymakers should focus on reducing air pollution and improving the efficiency of economic and health expenditures to maximize their benefits for LE.

## 3 Methodology and data

### 3.1 Model specification

This research aims to examine the impact of environmental degradation, GPR, institutional quality and technological innovation on LE. The model employed in this study is derived from earlier research conducted by literatures:


$$\begin{array}{l} {\mathrm{LE}}_{\mathrm{it}}=\vartheta_{0}+\vartheta_{1}\mathrm{CO}2-E_{\mathrm{it}}+\vartheta_{2}\mathrm{GP}R_{\mathrm{it}}+\vartheta_{3}IQ_{\mathrm{it}}\\ +\vartheta_{4}TI_{\mathrm{it}}+\vartheta_{5}\mathrm{GD}P_{\mathrm{it}}+e_{\mathrm{it}}\; \end{array}$$ (1)

In Equation 1, *LE, CO2-E, GPR, IQ, TI* and *GDP* represent life expectancy, Carbon emission, geopolitical risk, institutional quality, technological innovation and gross domestic product, respectively. Where, $e_{\mathrm{it}}$ denote the error term. LE is a dependent variable while *CO2-E, GPR, IQ, TI* and *GDP* are independent variable. To overcome the problem of data sharpness and Hetrocedasticity, we convert all the variables into natural logarithm except IQ.

### 3.2 Estimation strategy

#### 3.2.1 Cross sectional dependency

Cross-sectional dependency (CSD) arises when shocks in one country or unit spill over to others, creating interlinked outcomes. In panel data, ignoring this dependence can lead to biased and inconsistent estimates. Therefore, tests such as LM, CD, and bias-adjusted LM are employed to detect its presence. A common approach is Pesaran’s CD test, formulated as in the Equations 2, 3:


$$\mathrm{LM}=J\sum_{i=1}^{K-1}\sum_{j=i+1}^{K}{{\hat{\delta }}^{2}}_{\mathrm{ij}}\;$$ (2)

$$\mathrm{CD}=\sqrt{\frac{2}{N(N-1)}}[\sum_{i=1}^{K-1}\sum_{j=i+1}^{K}{{\hat{\delta }}^{2}}_{\mathrm{ij}}]\;$$ (3)

Where ${{\hat{\delta }}^{2}}_{\mathrm{ij}}$ correlation of residuals between nation *i* and nation *j.*

#### 3.2.2 Panel unit root test

To test for unit roots, this study applies both the Levin-Lin-Chu (LLC) and the Cross-sectionally Augmented IPS (CIPS) tests. In the context of the LLC test, the core hypothesis regarding the presence of a unit root in panel data is formulated as follows:


$$\Delta W_{\mathrm{it}}=\propto_{i}+\Phi_{i}W_{i,t-1}+\sum_{L=1}^{\emptyset 1}\emptyset_{i,1}\Delta W_{i,t-L}+\varepsilon_{i,t}$$ (4)

Where Equation 4, $W_{\mathrm{it}}$ , Δ , $\propto_{i}$ and $\emptyset_{i}$ represent the variable of interest, first difference, individual fixed effect, lagged differences. If H0:ϕ1=0 to test for the presence of a unit root. And H1:ϕ1<0 across all i to establish the absence of a unit root (60). The CIPS test, proposed by Pesaran (62), extends the Im-Pesaran-Shin (IPS) test by addressing CSD in panel data. It modifies the standard unit root equation by incorporating cross-sectional averages:


$$\Delta W_{\mathrm{it}}=\gamma_{i}+\emptyset_{i}W_{\mathrm{it}-1}+\alpha_{i}{\bar{W}}_{t-1}+\sum_{i=0}^{p}\delta_{i}\Delta {\bar{W}}_{t-1}+\sum_{i=1}^{p}\theta_{i}\Delta W_{\mathrm{it}-I}+e_{\mathrm{it}}\;$$ (5)

Where Equation 5, ${\bar{W}}_{t}$ mean cross-sectional average capturing CSD. The test evaluates whether $\emptyset_{i}=0$ (unit root) or $\emptyset_{i}<0$ (stationary) across panel units (24).

#### 3.2.3 Cs ARDL

The Cross-Sectional Autoregressive Distributed Lag (CS-ARDL) econometric method, developed by Chudik and Pesaran (63), was employed to examine the potential long- and short-run effects of CO2-E, GPR, IQ and TI on life expectancy. The CS-ARDL extends the standard ARDL approach by incorporating cross-sectional averages of variables and their lagged values to address CSD. The generic CS-ARDL model is presented in Equation 6, below:


$$LE_{i,t}=\varphi_{i}+\sum_{j=1}^{p}\gamma_{\mathrm{ij}}LE_{i,t-j}+\sum_{j=0}^{q}\alpha_{\mathrm{ij}}Z_{i,t-j}+\sum_{j=0}^{r}\beta_{\mathrm{ij}},{\bar{X}}_{t-1}+u_{\mathrm{it}}\;$$ (6)

This study adopts the CS-ARDL framework due to its strength in overcoming key econometric issues such as CSD, slope heterogeneity, and endogeneity, which are often inadequately addressed by conventional regression techniques (64). The model extends Pesaran’s (65) panel ARDL approach by treating the lagged dependent variable as weakly exogenous, allowing it to account for unobserved common influences across panel units. Instead of explicitly modeling these unobserved factors, the CS-ARDL approach uses cross-sectional averages of the observed variables and their lags to capture shared dynamics. CS-ARDL is preferred as it accommodates variables with mixed orders of integration [I(0) and I(1)], unlike CCEMG or AMG which require all variables to be I(1). It captures both short-run dynamics and long-run relationships simultaneously, while CCEMG and AMG focus only on long-run estimates. This method is particularly effective in panels where the time dimension exceeds the cross-sectional dimension (*T > N*), providing reliable estimates regardless of whether the variables are stationary or non-stationary, while also reducing sample selection bias. This choice avoids over-parameterization while ensuring an appropriate model fit. The model’s error correction form includes the error correction term (*ECT*), short- and long-run components, and cross-sectional averages for each variable, as shown in Equation 7, below:


$$\begin{array}{l} \mathrm{\Delta L}E_{i,t}=\varphi_{i}+\forall_{i}(LE_{i,t-1}-{\overset{´}{\theta }}_{i}Z_{i,t-1})+\sum_{j=1}^{p-1}{\gamma^{*}}_{\mathrm{ij}}\mathrm{\Delta L}E_{i,t-j}\\ +\sum_{j=0}^{q-1}{\alpha^{*}}_{\mathrm{ij}}\Delta Z_{i,t-j}+\sum_{j=0}^{r}\beta_{\mathrm{ij}},{\bar{X}}_{t-1}\\ +\sum_{j=1}^{p-1}{ℵ}_{j}{\bar{\mathrm{\Delta LE}}}_{t-1}+\sum_{j=0}^{q-1}\omega_{j}{\bar{\mathrm{\Delta Z}}}_{t-1}+u_{\mathrm{it}}\; \end{array}$$ (7)

Where, $\forall_{i}(LE_{i,t-1}-{\overset{´}{\theta }}_{i}Z_{i,t-1})$ , signifies the long-term relationship between LE and explanatory variables and ${\overset{´}{\theta }}_{i}$ shows long-term scalar elasticities. The term $\mathrm{\Delta L}E_{i,t}$ , and $\Delta Z_{i,t-j}$ shows the short-run dynamic of the dependent variable and regressors, respectively. $\bar{X}={\bar{\mathrm{LE}}}_{t-1},{\bar{Z}}_{t-1}$ is the long-run the cross-sectional means of the dependent factor ${\bar{\mathrm{LE}}}_{t-1}$ and model covariates ${\bar{Z}}_{t-1}$ (66).

**Figure 1:**
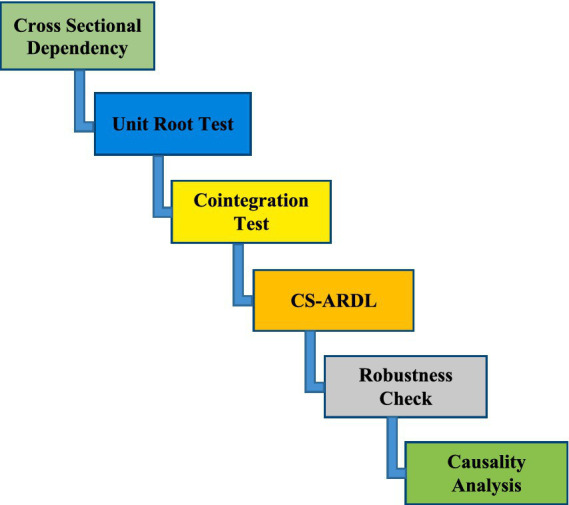
Estimation strategy.

The validation of the CS-ARDL estimation was assessed using the Fully Modified Ordinary Least Squares (FMOLS) and Dynamic Ordinary Least Squares (DOLS) estimators. These methods are widely recognized for their resilience in handling CSD, endogeneity, and other econometric challenges in panel data analysis. The FMOLS estimator corrects for serial correlation and endogeneity by modifying least squares regression, ensuring asymptotically unbiased estimates in the presence of cointegration. Meanwhile, the DOLS estimator enhances estimation accuracy by incorporating leads and lags of first-differenced explanatory variables, effectively addressing endogeneity and small-sample bias. Additionally, this study employed the Dumitrescu and Hurlin (67) panel causality test, which examines the causal relationships between the observed factors under the null hypothesis *(𝐻0)* of homogeneous non-causality. This approach allows for heterogeneity in causal relationships across cross-sectional units, making it suitable for dynamic panel data settings. By identifying directional causality among key variables, the results offer valuable insights for policymakers in designing regulatory frameworks that effectively address economic and environmental challenges. See Figure 1, estimation strategy.

### 3.3 Data

This study take four BRIC economies from 1993 to 2022, by using the six variables such as LE, CO2-E, GPR, IQ, TI and GDP. The data has been taken from World development indicator (WDI), policy uncertainty and world governance indicator (WGI). Table 1, shows the variable measurement. Technological innovation is measured using total patent applications, following the approach of Segbefia et al. (59). The IQ index was constructed based on six governance indicators commonly used in the literature (24). These indicators include (1) Control of Corruption (CC), (2) Government Effectiveness (GE), (3) Political Stability and Absence of Violence/Terrorism (PS), (4) Regulatory Quality, (5) Rule of Law, and (6) Voice and Accountability. Each indicator is measured on a scale ranging from −2.5 to +2.5, where lower values reflect weaker institutional performance and higher values reflect stronger institutional quality. Since these indicators are highly correlated, the IQI is derived using Principal Component Analysis (PCA). PCA reduces dimensionality by extracting components that capture the maximum variation across variables. The eigenvalues of the covariance matrix represent the explained variance of each component, and in most cases, the first principal component explains the largest share of variation. This first component is therefore used to construct the index. A key advantage of PCA is that the weights assigned to each indicator are determined objectively by the data, rather than arbitrarily (7).

**Table 1:** Variables measurement.

Symbol	Variables	Measurement	Data source
LE	Life expectancy	Birth, total (years)	WDI
CO2-E	CO2 emissions	per capita (t CO2-E/capita)	WDI
GPR	Geo political risk	Index	https://www.policyuncertainty.com/gpr.html
IQ	Institutional quality	Index	WGI
TI	Technological innovation	Total patient application	WDI
GDP	Gross domestic product	Per capita (constant 2015 US$)	WDI

## 4 Results and discussion

### 4.1 Descriptive statistics

Table 2 shows the descriptive statistics; the mean value of LE, CO2-E, GPR, IQ, TI and GDP are 71.026, 5.525, 156.595, −0.004, 175923.800 and 6100.025, respectively. The Standard deviation of the LE, CO2-E, GPR, IQ, TI and GDP are 3.993, 4.461, 120.493, 1.738, 379741.500 and 3300.240, respectively. The LE is positively correlated with all variables, especially TI, 0.58 and GDP (0.56), but weakly with CO2 emissions (0.10).

**Table 2:** Descriptive statistics.

	LE	CO2-E	GPR	IQ	TI	GDP
Mean	71.026	5.525	156.595	−0.004	175923.8	6100.02
Median	71.131	2.636	117.530	0.086	35,511	6941.4
Maximum	78.587	14.040	588.373	3.114	1,585,663	11555.9
Minimum	61.792	0.884	48.743	−4.663	0.000	695.2
Std. Dev.	3.993	4.461	120.493	1.738	379741.5	3300.2
Skewness	−0.135	0.611	2.088	−0.245	2.722	−0.398
Kurtosis	2.258	1.707	7.181	2.371	9.115	1.674
Jarque-Bera	2.517	12.784	141.166	2.571	270.8	9.668
Probability	0.284	0.002	0.000	0.277	0.000	0.008
Correlation matrix						
LE	1.000	0.100	0.513	0.211	0.587	0.565
CO2-E	0.100	1.000	0.372	−0.774	0.212	0.561
GPR	0.513	0.372	1.000	−0.106	0.481	0.576
IQ	0.211	−0.774	−0.106	1.000	0.117	−0.095
TI	0.587	0.212	0.481	0.117	1.000	0.278
GDP	0.565	0.561	0.576	−0.095	0.278	1.000

### 4.2 Cross-sectional dependency, panel unit root and Kao residual cointegration test results

The empirical analysis begins by examining CSD in the dataset, as addressing CSD is crucial for ensuring the reliability of panel estimations. The results of the CSD tests are presented in Table 3, where the null hypothesis (𝐻0) of cross-country independence is tested against the alternative hypothesis that cross-sectional units are interdependent. Based on the statistical significance of the *p*-values at the 1% level, the null hypothesis is strongly rejected across all tests, confirming the presence of CSD in the model residuals. This finding indicates that economic, environmental, or policy-related shocks in one BRIC country tend to have spillover effects on others, highlighting the interconnectedness of these economies.

**Table 3:** Cross-sectional dependence tests results.

Test	LE	CO2-E	IQ	TI	GDP
Breusch-Pagan LM	158.004^*^	94.903^*^	44.809^*^	99.203^*^	150.828^*^
Pesaran scaled LM	43.880^*^	25.664^*^	11.203^*^	26.905^*^	41.808^*^
Bias-corrected scaled LM	43.811^*^	25.595^*^	11.126^*^	26.837^*^	41.739^*^
Pesaran CD	12.559^*^	9.230^*^	−2.390^**^	9.792^*^	12.265^*^

The *, **, & *** represent 1, 5% & 10% level of significance.

To examine the stationarity of the variables, this study applied the Levin-Lin-Chu (LLC) test and the CIPS test. The results of these tests are presented in Table 4. According to the LLC test, only LE is stationary at the level, whereas the CIPS test indicates that GPR is stationary at the level. All other variables become stationary after first differencing, suggesting the presence of both I(0) and I(1) processes in the dataset. The Kao residual cointegration test confirms that at the 5% significance level, the null hypothesis of no cointegration is rejected. This indicates the presence of a long-run relationship among the variables in the model.

**Table 4:** Unit root test results.

Variables	LLC		CIPS	
	I(0)	I(1)	I(0)	I(1)
LE	−4.052^*^	−4.204^*^	−1.228	−3.432^*^
CO2-E	−0.151	−3.016^*^	−1.940	−2.432^**^
GPR	0.756	−6.698^*^	−2.583^*^	−4.630^*^
IQ	1.097	−4.352^*^	−1.229	−2.543^**^
TI	0.558	−11.037^*^	−1.071	−4.836^*^
GDP	4.126	−1.844^**^	−0.533	−3.832^*^
Kao residual cointegration test				
		t-Statistic	Prob.	
ADF		−2.176^**^	0.015	
Augmented Dickey-Fuller Test Equation		0.045		
HAC variance		0.066		

The *, **, & *** represent 1, 5% & 10% level of significance, CIPS critical value −2.57, −2.33 and −2.21.

### 4.3 CS-ARDL results

Table 5 shows the result of panel CS-ARDL. In the long run and short run, IQ, TI and GDP have a positive effect on LE, while CO2-E, and GPR negatively impacts the LE. The coefficients of CO2-E are −0.638 and −0.625, showing that a 1% increase in CO2-E reduces the LE by 0.638 and 0.625% in the long and short run. The reason for the negative sign. In the BRIC context, however, the adverse health effects of environmental degradation dominate because of Rapid industrial growth, high pollution intensity, and insufficient environmental regulations, exposing populations to higher levels of air pollution, respiratory diseases, and other health risks, outweighing the potential benefits of development. The outcome is consistent with a recent investigation conducted by Wang et al. (68), Wu et al. (71), Uddin et al. (12), Kolasa-Więcek & Suszanowicz (69), and Ebenstein et al. (70). According to Uddin et al. (12) research on SAARC economies, and found that CO2-E deters the LE. Pakistan’s LE has decreased due to CO2-E, according to Azam et al. (8) analysis. After looking at Pakistan’s annual statistics, Wang et al. (68) came to the conclusion that energy usage shortens life expectancy by causing environmental degradation. A recent research conducted by Wu et al. (71) looked at China’s urban population data between 2013 and 2017. According to the study, life expectancy may be raised by reducing air pollution. Between 1992 and 2016, air pollution had a detrimental impact on life expectancy in 20 European countries, according to Kolasa-Więcek & Suszanowicz (69). Air pollution reduces life expectancy, according to research done by Hill et al. (72), who looked at data from all 49 states in the US. Ebenstein et al. (70) conducted a study which found that life expectancy in Chinese cities was negatively impacted by air pollution at the city level. According to Younus et al. (73) the degradation of the environment causes serious diseases, poor health, and a shortened life span.

**Table 5:** CS-ARDL parameter estimates.

Variable	Long run			Short run		
	Coefficient	SE	*p*-value	Coefficient	SE	Prob.
CO2-E	−0.638^*^	0.093	0.000	−0.625^*^	0.160	0.000
GPR	−0.286^*^	0.087	0.002	−0.157^*^	0.029	0.000
IQ	0.739^*^	0.089	0.000	0.057^*^	0.009	0.000
TI	0.166^*^	0.037	0.000	0.001^*^	0.000	0.000
GDP	0.011^*^	0.004	0.007	0.699	0.604	0.564
ECM(−1)				−0.728^**^	0.346	0.032

The *, **, & *** represent 1, 5% & 10% level of significance. One lag selected for the CS-ARDL model based on the AIC.

The estimated coefficient of −0.286 and −0.157, showing that a 1% increase in GPR reduce the LE by 0.286 and 0.157% in the long and short runs. Thus, GPR involves political unrest, economic sanctions, and wars, can have a negative influence on LE and health in a number of ways such as (i) Direct violence: Political unrest and armed conflicts can result in direct violence that kills and injures people. (ii) Indirect violence: Conflict-related food shortages, housing displacement, and limited access to medical treatment can all have a negative impact on people’s health and longevity (iii) Disease outbreaks: Public health systems can be disrupted by political unrest and wars, which raises the danger of disease outbreaks and limits access to treatments and vaccinations (iv) Mental health: Prolonged exposure to stress, trauma, and violence as a result of geopolitical hazards can have a detrimental impact on mental health and increase the likelihood of mental illnesses including anxiety and depression. (v) Healthcare access: Geopolitical threats have the potential to restrict access to healthcare services, such as screening, treatment, and preventative care, which can have a negative impact on health and shorten life expectancy. The finding consistent with the line of Mahmud (27) and Wesemann et al. (29).

The estimated coefficient of IQ is 0.739 and 0.057, indicating that a 1 unit increase in institutional quality surges the LE by 0.739 and 0.057% in the long and short run. The reason of the positive sign in that Institutional quality improves LE by ensuring better governance, reducing corruption, and enhancing public service efficiency, leading to improved healthcare and social welfare. Strong institutions promote economic stability and equitable resource distribution, which supports access to clean water, sanitation, and medical care. The outcomes consistent with the line of Uddin et al. (7). Uddin et al. (7) examined the connection between IQ and LE in SAARC economies from 2002 to 2020 and they found that IQ rise the LE.

The coefficient of TI is also positive effect on LE, it shows that 1% surge the TI leads to raise the LE by 0.166 and 0.001% in long run and short run. Thus, TI enhances healthcare through advanced medical treatments, improved disease prevention, and better healthcare infrastructure, leading to longer life expectancy. The finding is consistent with the finding of Shaari et al. (60) and Muradov et al. (61). Shaari et al. (60) investigated the impact of green technology on LE in ASEAN-5 countries by Findings indicate that green technology significantly enhances LE. Muradov et al. (61) analyzed the relationship between air pollution and LE in the US, considering medical innovation, health expenditures, economic complexity, and government effectiveness. Using cointegration analysis and data from 1995 to 2019, the study finds that medical innovation positively affects LE.

The coefficient of GDP shows a positive effect on life expectancy (LE), indicating that a 1% increase in GDP raises LE by 0.011% in the long run. This positive relationship supports the existence of the Preston Curve, which links higher income to longer life expectancy. Similar findings were reported by Alhassan et al. (38), who showed that GDP per capita significantly enhances life expectancy across all income levels in developing economies. Higher GDP increases LE by funding better healthcare, nutrition, and living standards, reducing disease and mortality rates. It also supports infrastructure, education, and public services, creating healthier and safer environments for people. The finding is consistent with the finding of Azam et al. (8) and found that GDP raised the LE.

The ECM coefficient is −0.728, it means that 72.8% of any short-term deviation from the long-run equilibrium in LE is corrected each period (yearly). This implies a relatively fast adjustment process, suggesting that factors influencing LE quickly return to a stable long-run relationship over time.

### 4.4 Robustness analysis and causality analysis

For the robustness analysis (see Table 6), this study employs the FMOLS and DOLS estimators. Both methods confirm the same sign, although the coefficient magnitudes differ, which is attributed to the distinct estimation procedures. The empirical findings reveal that CO2-E and GPR have a significant negative impact on Life Expectancy LE. On the other hand, Institutional Quality, TI, and GDP positively influence LE. The Panel Causality Test shows in Table 7 that the Unidirectional causality was exposed from GPR to LE, IQ to LE, CO2-E to GPR, TI to CO2-E, GDP to GPR, TI to IQ, and GDP to IQ. The panel causality test reveals a unidirectional causality from GPR to LE, indicating that GPR significantly influences life expectancy. Higher geopolitical risks undermine stability, disrupt health systems, and negatively affect longevity. However, changes in LE do not influence geopolitical risk. The causality test results reveal a unidirectional causal relationship from IQ to LE. This implies that improvements in governance, rule of law, and institutional effectiveness lead to better health outcomes and longer life spans. However, the reverse causality from LE to IQ was not established, suggesting that health improvements alone do not directly strengthen institutions. Therefore, institutional reforms play a driving role in enhancing LE. While bi-directional causality exists between CO2-E and LE, LE and TI, GDP and CO2-E, IQ and GPR, TI and GPR. The panel causality results indicate a bidirectional causality between CO2-E and LE. This means rising CO2-E adversely affects health outcomes and longevity, while improvements in life expectancy, through economic growth and higher energy use, can also drive up emissions. The feedback relationship highlights the environmental health nexus.

**Table 6:** Robustness analysis.

Variable	FMOLS			DOLS		
	Coefficient	Std. error	Prob.	Coefficient	Std. error	Prob.
CO2-E	−0.739^***^	0.302	0.032	−0.781^*^	0.218	0.000
GPR	−0.133^*^	0.050	0.009	−0.148^**^	0.061	0.012
IQ	1.047^*^	5.527	0.003	4.621^***^	2.146	0.051
TI	0.301^*^	0.015	0.000	0.030^***^	0.016	0.077
GDP	0.136^*^	0.025	0.000	0.009^*^	0.002	0.000
Adj. R^2^	0.913			0.931		

The *, **, & *** represent 1, 5% & 10% level of significance.

**Table 7:** Panel causality test.

Null hypothesis	W-Stat.	Zbar-Stat.	Prob.
CO2-E ⇎ LE	20.686^*^	15.349	0.000
LE ⇎ CO2-E	7.081^*^	4.058	0.000
GPR ⇎ LE	5.221^**^	2.435	0.015
LE ⇎ GPR	4.145	1.563	0.118
IQ ⇎ LE	5.305^**^	2.377	0.018
LE ⇎ IQ	3.872	1.260	0.208
TI ⇎ LE	1.526	−0.551	0.582
LE ⇎ TI	28.338^*^	21.699	0.000
GDP ⇎ LE	47.872^*^	37.909	0.000
LE ⇎ GDP	8.201^*^	4.987	0.000
GPR ⇎ CO2-E	1.549	−0.540	0.589
CO2-E ⇎ GPR	9.061^*^	5.546	0.000
IQ ⇎ CO2-E	1.045	−0.944	0.345
CO2-E ⇎ IQ	3.673	1.105	0.269
TI ⇎ CO2-E	5.623^*^	2.848	0.004
CO2-E ⇎ TI	4.018	1.517	0.129
GDP ⇎ CO2-E	5.107^**^	2.420	0.016
CO2-E ⇎ GDP	9.282^*^	5.885	0.000
IQ ⇎ GPR	7.240^*^	3.842	0.000
GPR ⇎ IQ	7.731^*^	4.221	0.000
TI ⇎ GPR	7.356^*^	4.165	0.000
GPR ⇎ TI	7.747^*^	4.481	0.000
GDP ⇎ GPR	8.275^*^	4.909	0.000
GPR ⇎ GDP	2.490	0.222	0.824
TI ⇎ IQ	6.671^*^	3.442	0.001
IQ ⇎ TI	4.270	1.571	0.116
GDP ⇎ IQ	4.803^**^	1.986	0.047
IQ ⇎ GDP	1.421	−0.651	0.515
GDP ⇎ TI	3.083	0.741	0.459
TI ⇎ GDP	6.981^*^	3.975	0.000

The *, **, & *** represent 1, 5% & 10% level of significance.

## 5 Conclusion and policy recommendations

This study examined the impact of Environmental degradation and geopolitical risk on life expectancy in BRIC countries with the role of institutional quality and technological innovation in LE from 1993 to 2022. This study utilized the CSD, Panel unit root (LLC and CIPS), Kao Residual Cointegration test, CS- ARDL, FMOLS, DOLS and Panel causality estimators. The unit root test confirmed that there is mixed order of the data stationarity. The Cointegration test confirmed that there is long run relationship between the variables. The CS ARDL, FMOLS and DOLS estimates confirmed that CO2-E, GPR have negative effect on LE, while IQ and TI have positive effect on LE. The panel causality confirmed that Uni directional causality was expose from GPR to LE, IQ to LE, while bi directional causality exist between CO2-E and LE, LE and TI.

This study has several policy recommendation for the BRIC economies to enhance the LE: first, Governments should implement stricter emissions regulations, incentivize clean energy adoption, and promote carbon reduction policies. Investing in renewable energy sources, such as solar and wind power, can help reduce CO2-E and improve air quality, ultimately enhancing public health and increasing LE. Second, Governments should introduce carbon taxes and cap-and-trade systems to incentivize industries to lower their emissions and shift toward cleaner production methods. Third, Investing in diplomatic conflict resolution, mediation programs, and regional cooperation can help prevent conflicts and geopolitical tensions. Reducing wars and civil unrest ensures that healthcare services, infrastructure, and public health programs remain functional. Fourth, Strengthening border security, cybersecurity, and intelligence-sharing mechanisms can mitigate the risks of terrorism, political violence, and economic sabotage. A secure environment fosters stable economic growth and public service delivery, improving healthcare access and LE. Fifth, Governments should promote transparent governance, the rule of law, and democratic institutions to reduce political instability. Strong institutions ensure policy continuity, economic stability, and improved healthcare systems, which collectively enhance LE. Six, Institutional reforms should focus on universal healthcare coverage, improved regulatory frameworks, and equitable distribution of medical resources to ensure that all citizens, especially vulnerable populations, receive quality healthcare. A well-functioning healthcare system reduces mortality rates and promotes longer, healthier lives. Seventh, Governments should enforce transparent governance, accountability mechanisms, and anti-corruption policies to ensure efficient allocation of public resources, particularly in healthcare, sanitation, and social services. Reduced corruption leads to better healthcare infrastructure and improved access to medical services, ultimately increasing LE. Eight, Governments should allocate more funding to biomedical research, pharmaceutical innovation, and advanced medical equipment to improve disease prevention, early diagnosis, and treatment. Cutting-edge healthcare technologies can significantly reduce mortality rates and extend LE. Ninth, encouraging the adoption of clean energy, innovative infrastructure, and environmental monitoring systems helps reduce pollution, improve sanitation, and mitigate health risks. Sustainable technological innovations ensure healthier living conditions, leading to longer LE. Tenth, policymakers should prioritize reforms that enhance governance, healthcare system efficiency, and transparency. Targeted improvements in regulatory frameworks, anti-corruption measures, and public health management could directly translate into quantifiable health gains, improving LE across BRIC nations. Eleventh, since higher GPR significantly reduces life expectancy, policymakers should focus on strategies to reduce geopolitical tensions and enhance national stability. Diplomatic engagement, conflict prevention, and regional cooperation initiatives could lessen the adverse impact of GPR on population health.

This study has several limitations that provide opportunities for future research. First, the analysis focuses only on four BRIC countries and excludes South Africa due to the unavailability of GPR data. Future research can extend this study by incorporating South Africa once reliable GPR data becomes available, allowing for a more comprehensive understanding of the BRICS economies. Second, this study examines the impact of GPR, CO2-E, IQ, and TI on Life Expectancy but does not account for other political, social, and economic factors that may also influence LE. Future studies can explore additional variables such as income inequality, healthcare expenditure, education, and environmental policies to provide a more holistic view of the determinants of life expectancy. Third, this study utilized the CO2-E as a proxy for environmental degradation. While CO2-E is a widely accepted measure, it does not fully capture the complexity of environmental issues. Other important indicators such as particulate matter concentrations (PM2.5), ecological footprint, biodiversity loss, water pollution, and solid waste generation also provide critical insights into environmental degradation. Future studies should therefore adopt a multidimensional approach by incorporating these additional indicators, which would allow for a more comprehensive and nuanced understanding of the link between environmental degradation and life expectancy. Fourth, Limitation: This study used total patent applications as a proxy for technological innovation due to the unavailability of more specific indicators such as patents in medical technology classes or pharmaceutical R&D expenditure. Future studies may employ these more precise proxies if such data become available. Finally, this study employs CS-ARDL, FMOLS and DOLS estimators, but does not incorporate other advanced econometric methods such as Asymmetric CS-ARDL, Common Correlated Effects Mean Group (CCEMG), and nonlinear approaches. Future research can apply these advanced techniques to capture potential asymmetries and CSD, leading to more robust and nuanced findings.

## Data Availability

The raw data supporting the conclusions of this article will be made available by the authors, without undue reservation.
